# ORM1 Mediates Ln-IgG-Induced Podocyte Damage and Autophagy via the AMPK/mTOR Signaling

**DOI:** 10.1080/15476278.2025.2519614

**Published:** 2025-06-17

**Authors:** Jie Chen, Libin Zou, Lu Liu, Chunfeng Wu, Mi Hu

**Affiliations:** a Department of Nephrology, Wuhan Third Hospital, Wuhan, Hubei Province, China; b Department of Nephrology, Wuhan Asia General Hospital, Wuhan, Hubei Province, China; c Pediatric Cinic, Wuhan Third Hospital, Wuhan, Hubei Province, China; d Department of Urology Surgery, Guangdong Shunde Xinrongqi Hospital, Foshan City, Guangdong Province, China; e Pediatric Ward, Wuhan Third Hospital, Wuhan, Hubei Province, China

**Keywords:** AMPK/mTOR, autophagy, lupus nephritis, ORM1, podocytes

## Abstract

Podocyte damage is a central feature of lupus nephritis (LN), making the identification of potential therapeutic targets to prevent podocyte injury and improve treatment outcomes essential. ORM1 has been suggested as a significant candidate gene in LN. In this study, mouse podocytes were induced using Immunoglobulin G (IgG) extracted from lupus patients. To investigate the role of ORM1, ORM1 knockdown was performed, and the effects on podocyte viability and apoptosis were assessed using the cell counting kit-8 (CCK-8) assay and flow cytometry. Additionally, autophagy markers LC3II/I and p62 were measured by western blotting and immunofluorescence, and the expression of the AMPK/mTOR signaling pathway was evaluated using western blotting. The results showed an upregulation of ORM1 in the LN model. Upon stimulation with IgG from LN patients, ORM1 knockdown reversed the reduction in podocyte viability, decreased the apoptosis rate, and reduced the elevated levels of autophagy, followed by an increase in AMPK phosphorylation and a decrease in mTOR phosphorylation. In conclusion, these results suggest that ORM1 modulates the expression of autophagy-related components in podocytes through the AMPK/mTOR signaling pathway, thereby influencing podocyte damage in the LN model in vitro.

## GRAPHICAL ABSTRACT

**Figure:**
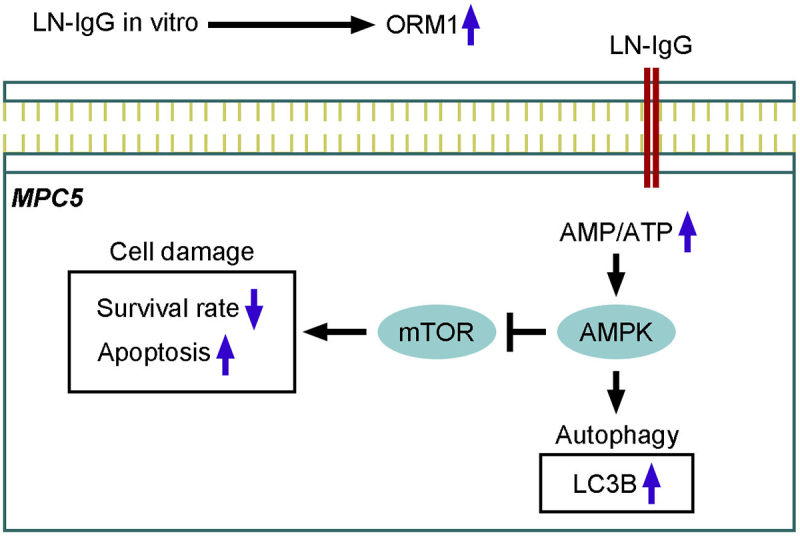

## Introduction

Systemic lupus erythematosus (SLE) is a group of autoimmune diseases that involve abnormalities at multiple stages of the immune system, leading to a diverse array of clinical symptoms and the production of various autoantibodies.^1^ In SLE, the immune system mistakenly recognizes self-antigens, which triggers the production of pathogenic autoantibodies and activation of type I interferon signaling, driving the development of the disease.^2^ Among the various manifestations of SLE, lupus nephritis (LN) is the most severe form of organ involvement and a type of glomerulonephritis.^3^ LN is strongly associated with increased morbidity and mortality in individuals with SLE, who generally have a poorer prognosis compared to those without nephritis, and can be related to both disease and treatment factors.^4^

Podocytes, the highly specialized epithelial cells in the kidney, play an essential role in maintaining selective glomerular filtration.^5^ The slit diaphragm complex, which includes the podocyte proteins podocin and nephrin, connects the foot processes to form a barrier that prevents the passage of solutes and proteins into the urine.^6^ Although numerous investigations have described the importance of podocyte injury in lupus glomerulonephritis, the underlying molecular mechanisms remain unclear. Therefore, a more in-depth understanding of podocyte damage in LN is essential for developing targeted therapeutic strategies.

Orosomucoid 1 (ORM1) has been reported to be a potential biomarker for the early identification of LN.^7^ It is an acute-phase protein that plays an important role in inflammatory responses and helps regulate immune and cellular stress responses.^8^ In mice, ORM2, another acute-phase protein, has been shown to directly enhance the synthesis of proinflammatory mediators and contribute to the development of chronic arthritis.^9^ ORM1 also promotes NFκB-mediated inflammation in vivo and plays a role in the progression of chronic graft rejection following renal transplantation. Additionally, ORM1 may serve as an indicator of psoriatic inflammation.^10^ As an acute-phase protein, ORM1 can exert systemic effects influencing renal function, and its role in regulating inflammation and immune responses may indirectly affect the kidneys.^11^ Based on these findings, we hypothesize that ORM1 could be involved in podocyte injury and repair, particularly as inflammation contributes to podocyte damage in various glomerular diseases.

In this study, we induced podocyte injury in vitro to replicate LN using IgG extracted from the serum of LN patients to investigate the specific effects of ORM1 on LN podocytes and explore the underlying mechanisms.

## Method

### Sample collection

Serum samples were collected from 20 LN patients and 10 healthy controls admitted to the Renal Immunology Department of Wuhan Third Hospital to extract and purify IgG. Informed consent was obtained from all participants, and the study was approved by the Ethics Committee of Wuhan Third Hospital (Approval no. KY2018–042).

### Extraction and determination of serum IgG concentration

Serum IgG was eluted, separated, and purified using a protein affinity chromatography column. The IgG concentration was determined following the manufacturer’s instructions (Elabscience Biotechnology Co. Ltd., Houston, TX, USA) after loading, washing, equilibration, and neutralization.^12^

### Cell culture and model establishment

The mouse podocyte cell line MPC5 was obtained from the Chinese Academy of Sciences Cell Bank and cultured in RPMI-1640 medium supplemented with 10% fetal bovine serum in a cell culture incubator at 37°C, with 5% CO_2_. To model LN in vitro, the cells were exposed to 1000 μg/mL lupus serum IgG for 6 hours, while the control group was treated with IgG from healthy patient serum.^12^

### Cell transfection

Before exposure to serum IgG for modeling, MPC5 cells were transfected with ORM1 mimic or negative control (NC) mimic (HZC05014, Genepharma, Shanghai). si-NC and si-ORM1 were transfected into cells using Lipofectamine® 2000 reagent (Thermo Fisher Scientific, USA) following the manufacturer’s protocol. After 48 hours, proteins were extracted from the cells to confirm the transfection efficiency. ORM1 sequence: forward: 5′-UUAUUGUACUCCUCGUUUCGA-3′, reverse: 5′-GAAACGAGGAGUACAUAAGU-3′.

### Cell counting kit-8 assay (CCK8)

After transfection, 5 × 10^3^ podocytes were exposed to lupus serum IgG for 6 hours, and following induction, 10 μL of CCK8 solution was added to each well, and the supernatant was discarded. The optical density (OD) at 450 nm was measured using a microplate reader after a 1-hour incubation period.

### Detection of apoptosis

Next, 5 × 10^4^ podocytes were exposed to lupus serum IgG for 6 hours. The cells were seeded into a 6-well plate, washed with cold PBS, and stained with 100 μL of binding buffer containing 10 μL of PI dye and 5 μL of Annexin V/FITC at room temperature for 15 minutes. Following staining, 400 μL of binding buffer was added, and apoptosis was analyzed using flow cytometry (Beckman Coulter, USA).

### Immunofluorescence

For this experiment, 5 × 10^3^ podocytes were exposed to lupus serum IgG for 6 hours and plated in a confocal culture dish. The cells were then fixed with 4% paraformaldehyde for 30 minutes and blocked for 20 minutes with 0.1% Triton X-100 in PBS. Afterward, they were blocked with 10% bovine serum albumin for 30 minutes. The cells were incubated with LC3B primary antibody (1:800, ab192890, UK) at 4°C for 1 hour. Subsequently, they were incubated with a fluorescent secondary antibody (1:200, 211061011, Beijing Zhongshan Jinqiao Biological Co. Ltd.) for 1 hour in the dark. For nuclear staining, DAPI was applied for 10 minutes, followed by the addition of an anti-fluorescence quencher. The stained cells were then observed and analyzed using a laser confocal microscope.

### Western blotting assay

After 6 hours of IgG treatment, the cells were collected, total protein was extracted using a protein extraction kit (Beyotime, China) following the manufacturer’s instructions, and the protein concentration was determined using a OneDrop device (Hangzhou, China). A total of 10 μg of protein was separated by SDS-PAGE and transferred to PVDF membranes (Millipore, USA) specific to the target protein’s molecular weight. The membranes were blocked for 1 hour at room temperature with 5% skim milk and incubated with primary antibodies against the candidate proteins for 12 hours at 4°C. After washing with PBS, the membranes were incubated with secondary antibodies at room temperature for 1 hour. Protein bands were detected using an ECL kit on a Tanon 4800 imaging system (Shanghai, China). The primary antibodies used were: ORM1 (ab134160, 1:5000), p62 (1:1000, ab207305), LC3-II/LC3-I (1:1000, ab192890), AMPK (1:1000, ab32047), p-AMPK (1:1000, ab133448), mTOR (1:1000, ab134903), p-mTOR (1:1000, ab109268), and GAPDH (ab9485, 1:1000). The secondary antibody used was goat anti-rabbit IgG (A0208, 1:10000, Beyotime, China).

### Statistical analysis

Data analysis was performed using GraphPad Prism 8.0 (GraphPad Software, San Diego, CA, USA) and SPSS 23.0 (IBM, Armonk, NY, USA). All data are presented as mean ± standard deviation. To compare two groups, the independent samples *t*-test was used, and one-way analysis of variance (ANOVA) was used to compare differences among multiple groups. *p* < 0.05 was considered statistically significant.

## Result

### ORM1 is highly expressed in LN in vitro models

**Figure 1.**
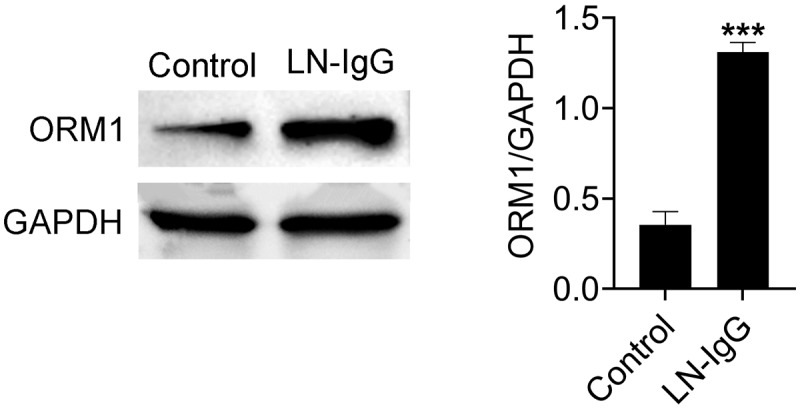
ORM1 is highly expressed in LN in vitro models. Expression levels of ORM1 in podocytes before and after treatment with LN-IgG. Values are presented as mean ± SD. *** *p* < 0.001 versus control group. *n* = 3. Each experiment was performed independently three times.

Autoantibody deposition in the glomeruli is a key factor contributing to renal impairment in SLE. To create an in vitro model of LN, we treated podocytes with IgG from LN patients (IgG-LN) for 6 hours. Western blot analysis revealed that podocytes exposed to IgG-LN exhibited significantly higher levels of ORM1 expression compared to the control group (*p* < 0.001) (Figure 1), indicating that ORM1 is upregulated in LN in vitro models.

### Knockdown of ORM1 alleviates LN-IgG-induced damage in podocytes

**Figure 2.**
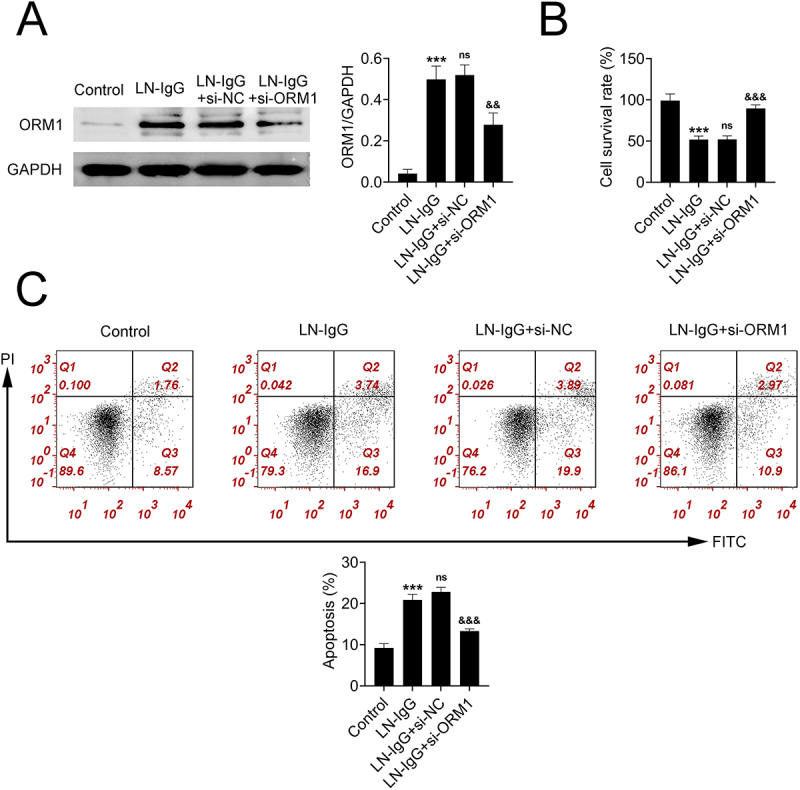
Knockdown of ORM1 alleviates LN-IgG-induced damage in podocytes. (A) ORM1 protein expression in podocytes before and after transfection with si-ORM1. (B) CCK8 assay measuring podocyte survival. (C) apoptosis rate of podocytes detected by flow cytometry. Values are presented as mean ± SD. *** *p* < 0.001 versus control group. ^&&^ *p* < 0.01, ^&&&^ *p* < 0.001 versus LN-IgG + si-NC group. *n* =3. Each experiment was conducted independently three times.

To further investigate the role of ORM1 in LN-induced podocyte damage, the expression of ORM1 was knocked down. As shown in Figure 2A, ORM1 expression was successfully reduced in the IgG-LN + si-ORM1 group (*p* < 0.01). In comparison to the control group, the apoptosis rate in podocytes exposed to IgG-LN was markedly increased, and their survival rate was significantly decreased (*p* < 0.001) (Figure 2B,C). However, ORM1 knockdown was able to reverse these detrimental effects of LN-IgG, reducing podocyte damage (*p* < 0.001) (Figure 2B,C). These results suggest that ORM1 mediates podocyte damage induced by LN-IgG.

### Knockdown of ORM1 alleviates LN-IgG-induced autophagy in podocytes

**Figure 3.**
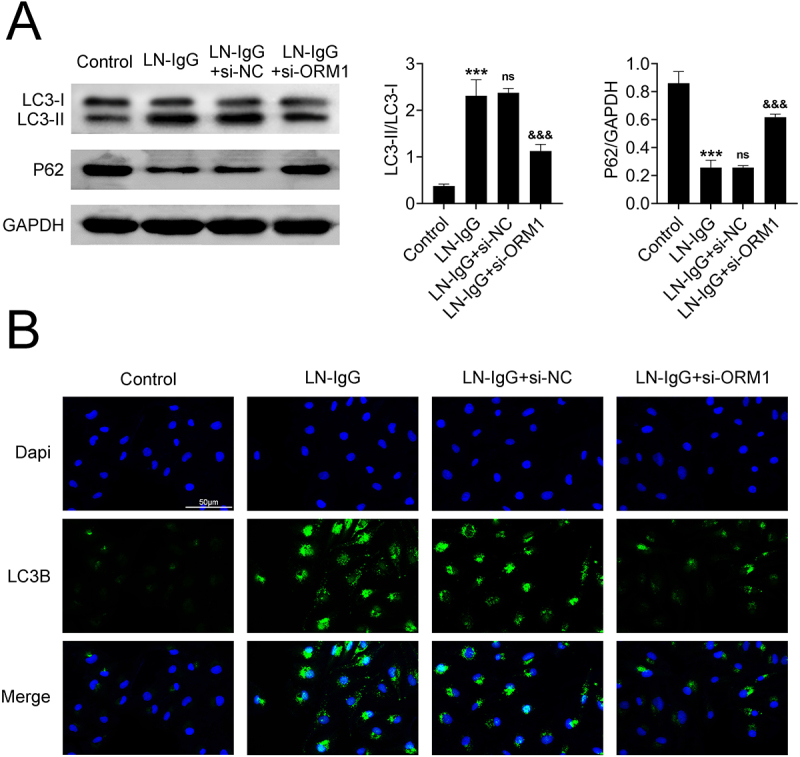
Knockdown of ORM1 alleviates LN-IgG-induced autophagy in podocytes. (A) expression of LC3-I/II and p62 proteins in podocytes before and after transfection with si-ORM1. (B) immunofluorescence detection of LC3B fluorescence intensity in podocytes before and after transfection with si-ORM1. Values are presented as mean ± SD. *** *p* < 0.001 versus control group. ^&&&^ *p* < 0.001 versus LN-IgG + si-NC group. *n* = 3. Each experiment was conducted independently three times.

Next, we investigated the role of ORM1 and autophagy in podocyte damage. In the LN-IgG-induced podocyte model, we observed a significant increase in the LC3II/LC3I ratio and a marked decrease in p62 expression, as shown by both Western blotting and immunofluorescence analysis (*p* < 0.001) (Figure 3A,B), and these effects could be reversed by knocking down ORM1 (*p* < 0.001) (Figure 3A,B). Together, these findings suggest that the downregulation of ORM1 reduces podocyte damage and autophagy induced by LN-IgG.

### Knocking down ORM1 regulates the AMPK/mTOR signaling pathway

**Figure 4.**
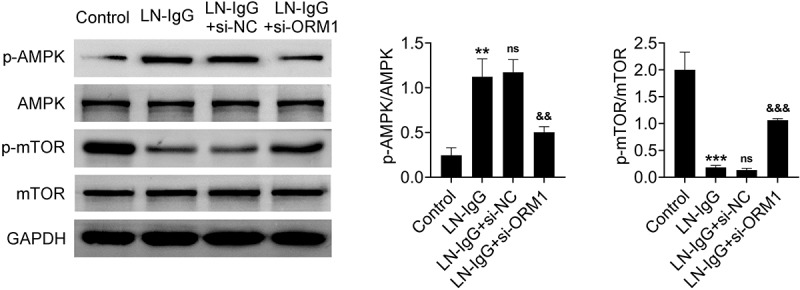
Knocking down ORM1 inhibits the AMPK/mTOR signaling pathway. Western blot analysis of AMPK, p-AMPK, mTOR, and p-mTOR protein expression in podocytes. Values are presented as mean ± SD. ** *p* < 0.01, *** *p* < 0.001 versus control group. ^&&^ *p* < 0.01 versus LN-IgG + si-NC group. *n* = 3. Each experiment was conducted independently three times.

To determine the molecular mechanisms underlying ORM1-mediated podocyte damage, we investigated the AMPK/mTOR signaling pathway. Western blot analysis revealed that phosphorylation of AMPK was increased, while phosphorylation of mTOR was decreased in LN-IgG-treated podocytes. In addition, these effects could be reversed following ORM1 knockdown (Figure 4). Based on these, we can suggest that the AMPK/mTOR pathway is a downstream effector of ORM1 in LN.

## Discussion

LN is one of the most severe manifestations of SLE, which is a prototypical autoimmune disease characterized by the production of numerous autoantibodies, complement activation, and immune complex deposition, all of which contribute to tissue and organ damage.^13,14^ Despite extensive research, the complete molecular pathways underlying both SLE and LN remain unclear. In this study, we used IgG to induce podocyte injury and autophagy abnormalities, mimicking LN in vitro.^12^ In this present study, our results demonstrate that LN-IgG can induce both podocyte injury and autophagy dysfunction, which aligns with the findings of previous studies by Yu et al.^15^

There have been several reports linking immune-related disorders to ORM1. For example, bioinformatics analyses have shown that ORM1 is differentially expressed in bone marrow from patients with immune thrombocytopenia, and is involved in the activation of immune functions and the TNF-α signaling pathway.^16^ ORM1 has also been identified as a potential biomarker for distinguishing between latent and active tuberculosis.^17^ Additionally, ORM1 has been proposed as a novel biomarker for early detection of LN.^7^ Collectively, these reports suggest that ORM1 expression is elevated in LN.

Autophagy also plays a crucial role in the development of LN,^18^ and functions as a double-edged sword, with both excessive and insufficient autophagy having physiological consequences for cells.^19^ It has been established that autophagy contributes to podocyte damage in LN. Studies have shown that autophagy levels are significantly elevated in renal tissue and blood samples from LN patients, correlating with extensive podocyte damage.^15,20^ Previous research has demonstrated that LN-IgG activates several signaling pathways, including the Cyr61 pathway, which is involved in the regulation of autophagy.^12^ Additionally, ORM1 has been shown to interact with various inflammatory mediators and signaling molecules that regulate autophagy.^21^ Based on these findings, we hypothesize that ORM1 contributes to the pathophysiology of renal disease by modulating autophagy disruptions in LN-IgG-induced podocyte injury.

A study by Qi et al. showed that vitamin D can reduce aberrant autophagy, protecting podocytes from autoantibody-induced damage in LN.^15^ Our findings are consistent with their findings, as we observed that ORM1 knockdown can also protect podocytes from damage in LN by lowering autophagy levels. Specifically, ORM1 knockdown increased p62 levels in IgG-treated podocytes, decreased the LC3II/LC3I ratio, and reduced the fluorescence intensity of LC3B. Based on these results, we further explored how ORM1 affects the proliferation of podocytes treated with IgG, and flow cytometry and CCK8 assays revealed that ORM1 can counteract the suppression of podocyte proliferation induced by IgG.

The AMPK-mTOR signaling pathway is a well-known regulator of autophagy.^22^ The inhibitory role of mTOR is thought to be associated with nutritional signals that regulate autophagy,^23^ and excessive activation of the mTOR pathway is crucial for podocyte damage, as it disrupts energy homeostasis, with AMPK serving as a sensor of cellular energy status.^24^ Herein, our results showed that in LN-IgG-induced podocytes, AMPK phosphorylation was upregulated while mTOR phosphorylation was downregulated. In addition, the knocking down of ORM1 could reverse this effect. The expression levels of mTOR in LN have been shown to vary based on disease severity, cell types, and immune response differences.^25^ However, future studies should further investigate the interaction between these factors to better understand mTOR’s role in LN and identify more precise targets for clinical treatment.

In LN patients, extensive immune complex deposition in renal tissue can activate local immune responses, leading to podocyte injury. Inflammatory mediators and cytokines can further exacerbate podocyte damage.^26^ ORM1 may indirectly protect podocytes by regulating immune cell function and alleviating inflammation.^7^ Future in vivo experiments could aim to explore the immune system and inflammatory responses more comprehensively.

## Conclusion

In conclusion, ORM1 could be a promising molecular marker for the diagnosis and treatment of LN as it can regulate the expression of autophagy components in podocytes through the AMPK/mTOR signaling pathway, thus influencing podocyte damage in the LN model in vitro.

## Data Availability

The authors declare that all data supporting the findings of this study are available within the paper and any raw data can be obtained from the corresponding author upon request.
